# Reproductive and environmental exposures and the breast cancer risk in Taiwanese women

**DOI:** 10.1038/s41598-021-95290-2

**Published:** 2021-08-02

**Authors:** Hui-Chen Wu, Hwai-I. Yang, Po-Han Lin, Chien-Jen Chen, Regina M. Santella, Mary Beth Terry

**Affiliations:** 1 Department of Environmental Health Sciences, Mailman School of Public Health of Columbia University, New York, NY 10032 USA; 2 Herbert Irving Comprehensive Cancer Center, Columbia University Medical Center, New York, NY USA; 3 Genomics Research Center, Academia Sinica, Taipei, Taiwan; 4 Institute of Clinical Medicine, National Yang–Ming University, Taipei, Taiwan; 5 Graduate Institue of Medicine, College of Medicine, Kaohsiung Medical University, Kaohsiung, Taiwan; 6 Biomedical Translation Research Center, Academia Sinica, Taipei, Taiwan; 7 Department of Medical Genetics, National Taiwan University, Taipei, Taiwan; 8 Graduate Institute of Epidemiology and Preventive Medicine, College of Public Health, National Taiwan University, Taipei, Taiwan; 9 Department of Epidemiology, Mailman School of Public Health of Columbia University, New York, NY USA

**Keywords:** Breast cancer, Biomarkers

## Abstract

Breast cancer (BC) incidence is increasing around the globe, including in Taiwan, though the cause of the increasing incidence is less clear. We followed up 11,296 Taiwanese females who did not have BC at baseline, and ascertained new invasive BC (N = 351) through data linkage to the National Cancer Registry from 1991 to 2018 to examine whether reproductive, lifestyle and environmental risk factors including polycyclic aromatic hydrocarbons (PAH) were associated with BC risk. We conducted a nested case–control study using baseline blood available from a total of 305 women with BC and 598 women without BC matched on time in cohort. We examined the association of PAH-albumin adducts and BC risk using conditional logistic regression models. Age at menarche (HR 0.6 (95% CI 0.5–0.9) for ≥ 15 vs. < 13 years) and multiparity were associated with BC risk (HR 2.0 (95% CI 1.4–2.8), 2.8 (1.9–4.2), and 2.4 (1.0–5.0) for 3–4, 1–2 and 0 live birth, compared with women ≥ 5 births). PAH-albumin adducts were not associated with BC risk. Given the increasing BC incidence in Taiwan, there is a need to identify environmental factors that are important to this population.

## Introduction

Breast cancer (BC) is the most common non-skin cancer malignancy in women worldwide, including Asia^1^. Although, historically the incidence of female BC in Asian countries is lower than that in western countries, the incidence is rising among Asian countries^2^. In Taiwan, the annual percent change (APC) for women under 50 is 4.71 (4.20, 5.22). There was a 2.7-fold difference in age-standardized incidence rates from 1990 to 2017^3^. This compares to an APC of 0.53 (0.29–0.78) in women under 50 in the U.S.^4^. Moreover, the peak age of onset in Western countries is 60–70 years, while in Asia it is 40–50^5^. Using a cancer registry, Shen et al*.* reported that changes in the reproductive pattern might be associated with the increase in the younger generation of Taiwanese women^6^. Environmental exposure such as air pollution is also suggested as a risk factor in Asian women^7^. Polycyclic aromatic hydrocarbons (PAHs), possible human carcinogens, are ubiquitous pollutants caused by incomplete combustion of various materials, including diesel fuel and tobacco^8^. Recently, we conducted a prospective study of women from the New York site of the BC Family Registry and reported that detectable levels of PAH-albumin adducts in blood was associated with a twofold increased BC risk compared with women with non–detectable levels^9^. The association was even stronger among women with higher absolute BC risk^9^.

In the present population-based long-term prospective study, we followed up a total of 11,296 Taiwanese females who did not have BC at study entry. The goal of this study was to understand the epidemiology of BC and examine the association of reproductive risk factors in BC. We also used a prospective nested case–control study design to examine the association of PAH-albumin adducts in baseline bloods and BC risk.

## Results

Table 1 presents the baseline characteristics for the total population at risk and for cases. Higher level of education was associated with a twofold increased risk (HR 2.2, 95% CI 1.1–4.3 for high school degree vs. never/elementary school). The prevalences of smoking and alcohol drinking were low in the cohort female participants. However, alcohol drinking was associated with an elevated, but not statistically significant, risk (HR 2.4, 95% CI 0.9–5.7). The prevalence of women whose mother had BC is less than 1%. The majority of women had menarche at age above 15 years and had 5 or more pregnancies. The HR for women with menarche at age of 15 and above was 0.6 (95% CI 0.5–0.9). Compared to women who had 5 or more pregnancies, the HR for women who had 3–4 pregnancies was 1.4 (95% CI 1.1–1.7). The HRs were 1.8 (95% CI 1.3–2.6) and 1.8 (95% CI 0.9–3.5) for women with 1–2 or 0 pregnancies. Compared to women who had 5 or more live births, the HR for women who had 3–4 live births was 2.0 (95% CI 1.4–2.8). The HRs were 2.8 (95% CI 1.9–4.2) and 2.4 (95% CI 1.1–5.0) for women with 1–2 or 0 live births. Birth control was not associated with BC. The associations were consistent across different birth cohorts (data not shown).

**Table 1:** The hazard ratios (HR) of selected variables for breast cancer, the cancer screening program cohort.

	Population at risk/cases N = 11,296/351 N (%)	Person-years (278,301)	Age adjusted HR
**Birth cohort**			
1918–1940	4138/97	95,253	1.0
1941–1950	3106/105	78,434	1.3 (0.9–1.7)
1951–1962	4052/149	104,613	1.4 (1.1–1.8)
**Age at recruitment (years)**			
< 40	3458/132	89,452	1.0
40–50	3207/100	81,347	0.8 (0.6–1.1)
50–60	3379/96	80,750	0.8 (0.6–1.1)
60–70	1252/23	26,751	0.6 (0.4–0.9)
**Education**			
Never/elementary	8105/198	196,564	1.0
High school	3032/144	77,662	1.9 (1.4–2.4)
Colleague and above	159/9	4,074	2.2 (1.1–4.3)
**Cigarette smoking**			
No	11,171/348	275,367	1.0
Yes	125/3	2,933	0.8 (0.3–2.5)
**Alcohol consumption**			
No	11,226/346	276,638	1.0
Yes	70/5	1,662	2.4 (0.9–5.7)
**BMI (kg/m**^**2**^**)**			
< 24	6046/189	151,213	1.0
24–27	3194/105	78,025	1.1 (0.9–1.5)
≥ 27	2056/57	49,062	1.0 (0.7–1.4)
**Mother had breast cancer**			
No	11,253/349	277,241	1.0
Yes	43/2	1,059	1.5 (0.4–5.8)
**Age at regular menarche (year)**			
< 13	1436/61	35,914	1.0
13–15	4101/149	102,454	0.9 (0.6–1.2)
≥ 15	5759/141	139,931	0.6 (0.5–0.9)
**No. of pregnancy**			
≥ 5	6156/151	148,980	1.0
3–4	4008/146	100,840	1.4 (1.1–1.7)
1–2	935/45	23,552	1.8 (1.3–2.6)
0	197/9	4,929	1.8 (0.9–3.5)
**No. of live birth**			
≥ 5	3193/51	74,759	1.0
3–4	5892/200	147,465	2.0 (1.4–2.8)
1–2	1978/91	50,265	2.8 (1.9–4.2)
0	233/9	5,809	2.4 (1.1–5.0)
**Menopausal status at enrolment**			
Premenopausal	7343/264	187,712	1.0
Postmenopausal	3953/87	90,588	0.8 (0.6–1.1)
**Oral contraceptives use**			
Never	7866/233	192,518	1.0
Ever	3430/118	85,782	1.1 (0.9–1.5)
**Intrauterine devices (IUDs) use**			
Never	4675/140	114,673	1.0
Ever	6621/211	163,627	1.1 (0.9–1.3)

*BMI* body mass index, *HR* hazard ratio.

### Cumulative hazard risk

**Figure 1:**
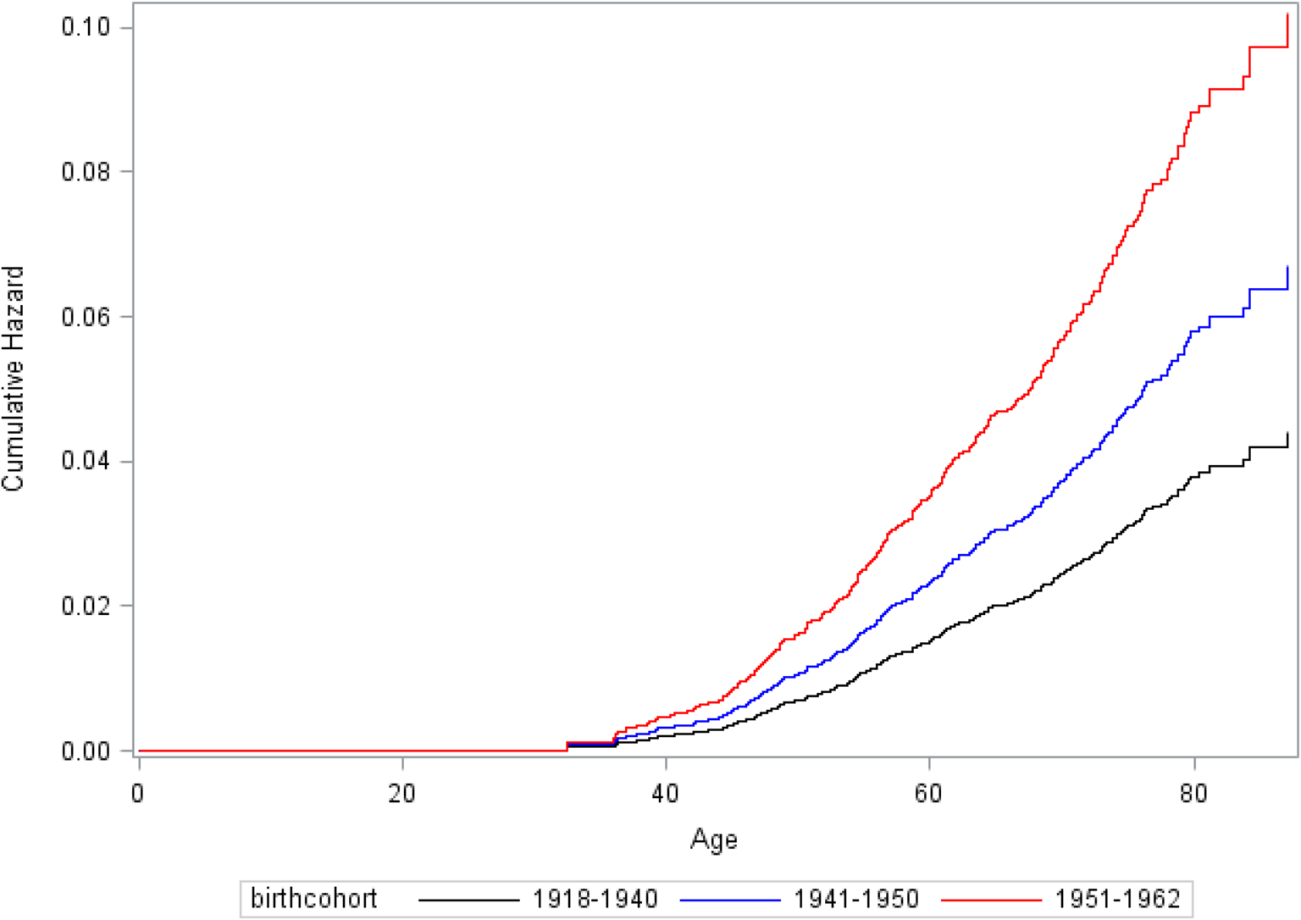
The cumulative hazard of breast cancer in Taiwanese women by different birth cohort.

The cumulative hazard was 0.04 (95% CI 0.03–0.05) for the birth cohort of 1918–1940 (Fig. 1). The cumulative hazards were 0.07 (95% CI 0.05–0.09) for birth cohort of 1941–1950 and 0.10 (95% CI 0.08–0.13) for birth cohort of 1951–1962.

Table 2 presents the association of PAH-albumin adducts and BC risk. We did not see any significant difference in the PAH-albumin adducts and BC risk overall, or by selected risk factors.

**Table 2:** The association of PAH-albumin adducts and breast cancer risk by selected risk factors, the cancer screening program cohort.

	Cases	Matched controls	Age adjusted OR (95% CI)
	N = 305	N = 598	
	No	No	
**PAH-Alb**			
Above versus below the median	135/170	299/299	0.8 (0.6–1.0)
**BMI < 25**			
PAH**-**Alb above versus below the median	58/87	140/173	0.8 (0.5–1.4)
**BMI ≥ 25**			
PAH**-**Alb above versus below the median	66/73	130/103	0.8 (0.4–1.3)
**Age at baseline < 45**			
PAH**-**Alb above versus below the median	58/98	130/171	0.8 (0.5–1.2)
**Age at baseline ≥ 45**			
PAH**-**Alb above versus below the median	66/62	140/105	0.8 (0.5–1.3)
**Premenopausal status at recruitment**			
PAH**-**Alb above versus below the median	84/127	169/210	0.8 (0.6–1.2)
**Postmenopausal status at recruitment**			
PAH**-**Alb above versus below the median	40/33	101/66	0.7 (0.4–1.4)

*Alb* albumin, *OR* odds ratio, *PAH* polycyclic aromatic hydrocarbons.

## Discussion

Overall our findings in the cohort analysis of Taiwanese women found only a small number of BC cases had a mother with BC. We also did not observe any association of ever oral contraceptive use with BC risk. Consistent with those from Western countries^10^ and other East Asian countries such as China and Korean^11–14^, we observed that selected reproductive factors were associated with BC in Taiwanese women: older age onset of menarche and multiparity were negatively associated with BC. The lack of an association with BC family history in this Taiwanese population and the large difference in APC across the countries, suggests that other risk factors such as lifestyle, reproductive factors or environmental exposures might be important^15^.

Although we did not observe a birth cohort difference in the association of reproductive factors and BC risk, the patterns of reproductive factors have changed in Taiwanese women. Similar to other countries, there has been a decrease in the age at menarche in the younger generation of Taiwan^16^. Among 214 adolescent girls, there was a 1.5 year difference in age at menarche between mothers and daughters, and a 2.1 year difference between grandmothers–granddaughters^16^. The fertility rate in Taiwan also decreased from 1.76 in 1991 to 1.15 in 2018 and the fertility rate in 2021 is only 1.07 which is the lowest fertility rate ever observed in Taiwan^17^. It is important to understand how much of the increase in the incidence of BC in Taiwan can be explained by the change in the reproductive factors. More studies are also needed to quantify the impact of changes in these reproductive factors on future BC incidence rate in Taiwan and compare the finding with other Asian countries. For example, the incidence of breast cancer is increasing in China^18^. Potential reasons for this increase may be related to substantially decline in the age of menarche and fertility rate^19,20^. According to a national survey^21^, the prevalence of both smoking and alcohol use are very low in Taiwanese women. Although the prevalences of smoking and alcohol use were low in our study participants, we observed an elevated increase in BC from alcohol consumption although this risk was not statistically significant. A recent survey of adolescents showed the prevalence of peer drinking in females was 49%^22^. Thus, the number of BC attributed to alcohol use would be expected to increase in the future.

Measuring urinary PAH metabolites, a biomarker of short-term PAH exposure, a nested case–control study in the Shanghai Women’s Health Study also reported no association between PAH exposure and BC risk in Chinese women^23^. We also did not observe an association between PAH-albumin adducts and BC risk. However, both our and the Shanghai Women’s Health Study were unable to time the exposure measurements to a window of susceptibility and were also unable to control for underlying genetic susceptibility^23^. The influence of environmental exposure on BC risk may be greater during several windows of susceptibility such as puberty^24^. Moreover, the effect of PAH exposure on cancer risk may vary with an individual’s ability to detoxify or eliminate the contaminant as well as DNA repair capacity. For example, we previously reported that polymorphisms in DNA repair genes modulate individual BC susceptibility related to exposure to PAHs^25,26^. In addition, we also found individuals with greater BC risk score based on a risk model that uses pedigree information were more vulnerable to PAH exposure^9^. As the prevalence of women whose mother had BC was low in our population, we were not able to examine the association of PAH exposure on BC risk stratified by family history of BC.

Although we did not observe an increased risk of BC associated with PAH-albumin adducts, other environmental exposures such as DDT and perfluoroalkly substances have been reported to be associated with BC in Taiwan^27,28^. In our systematic review of epidemiological literature on environmental exposure and BC risk, we concluded that studies enriched for women at higher BC risk through family history, younger age of onset and/or genetic susceptibility consistently support an association between an environment exposure and BC risk^29^.

A limitation of our study includes the lack of information on breast cancer molecular subtype. A hospital study examining the distribution of molecular subtypes of breast cancer in Taiwan reported that luminal A was the major subtype contributing to 61% of cases, following by basal-like (13%), HER-2+/ER−-(12%), luminal B (11%)^30^. It is unclear if the associations of reproductive factors differ by different subtypes in Taiwanese females. A case–control study conducted in China reported that late menopause, and lack of breastfeeding appear to increase risk of both luminal and ER-PR-tumors, while early menarche and nulliparity mainly impacted luminal tumor risk^31^. In addition, these associations were not impacted by menopausal status. Another limitation of our cohort analysis is that the epidemiologic information was collected at recruitment and not updated during the follow-up. Some of the reproductive information such as number of pregnancies and family history of breast cancer might change over time. In addition, PAH-albumin adduct level is a single measurement, and may not represent long-term PAH exposure from all exposure routes. The major strength of our study is that PAH-albumin adducts were measured in blood samples collected prior to cancer diagnosis. This rules out the possibility that the biomarker levels were due to metabolic changes associated with an already existing cancer.

In summary, we found no statistically significant association between PAH-albumin adducts and BC risk in Taiwanese women but similar reproductive risk factors as found in Western women. There is a need to understand what are the clinical drivers of the increase in BC risk in Taiwan and identify environmental factors that are important to this population.

## Materials and methods

### Study subjects

We used female participants from the community-based Cancer Screening Program cohort recruited in Taiwan. The cohort characteristics and methods of screening and follow-up have been described in detail previously^32^. Briefly, individuals who were between 30 and 65 years old and lived in seven townships in Taiwan with no history of cancer were recruited between 1991 and 1992 with a total of 11,973 males and 11,847 females. Participants were personally interviewed based on a structured questionnaire regarding epidemiological information and donated a 10 mL fasting blood sample at recruitment. Biospecimens were transported on dry ice to a central laboratory at the National Taiwan University and stored at − 80 °C until transport to Columbia University for analysis. Epidemiological information included socio-demographic characteristics, reproductive factors (female participants only), habits of alcohol intake and cigarette smoking, and mother’s BC status. Habitual cigarette smoking was defined as having smoked > 4 days/week for at least 6 months. Information about duration and intensity was also obtained. Habitual alcohol intake was defined as drinking alcohol containing products > 4 days/week for at least 6 months. Women who had no menstrual flow for 12 months were considered as postmenopausal and the rest were considered as premenopausal. Anthropometric measurements including height, weight, and hip and waist circumference were recorded using standardized protocols during the interview.

We ascertained newly developed female breast cancer (n = 365) among a total of 11,847 female participants by computerized data linkage with the National Cancer Registry from January 1, 1991, through December 31, 2018. For the cohort analysis, we used information from 11,296 female participants including 351 cases who completed questionnaire information. Among those 365 incident breast cancer cases, there were three cases were diagnosed within 1 year after recruitment. However, there three cases were not included in the cohort analysis as they did not completed questionnaire. All 351 cases were all diagnosed with breast cancer at least 1 year after baseline.

For the nested case–control study design, we used pre-diagnostic blood available from 305 cases for PAH-albumin adduct measurement. For controls, we randomly selected 598 women from prospective cohort subjects who were not affected with BC through the follow-up period by matching to each case by age (± 5 years), residential township, date of recruitment (± 6 months) and follow up time.

This study was approved by Columbia University’s Institutional Review Board (AAAB7226) as well as the Research Ethics Committee of the College of Public Health, National Taiwan University (AS-IRB01-18065), which conforms to the STROBE GUIDELINE for observation studies. All methods were performed in accordance with the relevant guidelines and regulations. Written informed consent was obtained from all subjects and strict quality controls and safeguards were used to protect confidentiality^33^.

### PAH-albumin adducts

We measured plasma PAH-albumin adducts using a competitive enzyme linked immunosorbent assay with monoclonal antibody 8E11 which we generated^34^. This antibody recognizes benzo(a)pyrene diolepoxide tetrols and related PAH metabolites^32^. All other reagents including secondary goat anti-mouse antiserum and *p*-nitrophenylphosphate were from Sigma-Aldrich. Four 96 well plates were run each day with one pooled quality control sample assayed in duplicate on each plate. The intra and inter-batch CV were 17.1% and 23.4%. Values are expressed as fmol of PAH per mg albumin.

### Statistical methods

#### Cohort analysis

Follow-up (in years) was considered as the time interval between the study entry and the earliest of these endpoints: date of BC diagnosis, date of death, or end of follow-up in the absence of BC diagnosis (December 31, 2018), whichever came first. To estimate the effect of various variables on the hazard of BC, we used Cox proportional hazards regression models using PROC PHREG procedure of SAS adjusting for age to calculate hazard ratios (HRs) and their 95% confidence intervals (CIs). We used follow-up time as the time scale and Schoenfeld’s global test to test the assumption of proportional hazards. For the figure, we used age as time scale to present the cumulative hazard by different birth cohort.

#### Nested case–control study analysis

We considered PAH-albumin adduct levels as a continuous variable and dichotomized into a single binary variable (above the median level vs. below the median level of controls). PAH-albumin adduct levels were natural log-transformed to normalize the distribution. We used the χ^2^ test for categorical variables and ANOVA for continuous variables to assess the difference of selected characteristics between cases and controls. After descriptive analyses, we used conditional logistic regression based on the matched sets (1:2 age matched) to estimate BC risk. All analyses were performed with SAS software 9.2 (SAS Institute, Cary, NC, USA). All statistical tests were based on two-tailed probability.

## Abbreviations

APC: Annual percent change
BC: Breast cancer
CSP: Cancer Screening Program
CI: Confidence interval
PAH: Polycyclic aromatic hydrocarbons
